# Telocytes are major constituents of the angiogenic apparatus

**DOI:** 10.1038/s41598-021-85166-w

**Published:** 2021-03-11

**Authors:** Soha A. Soliman

**Affiliations:** Department of Histology, Faculty of Veterinary Medicine, South Valley University, Qena, 83523 Egypt

**Keywords:** Cell biology, Developmental biology

## Abstract

The current study investigated role of telocytes (TCs) in angiogenesis during embryonic development of quail using immunohistochemistry (IHC), transmission electron microscopy (TEM), and scanning electron microscopy (SEM). The angiogenic apparatus consisted of TCs, endothelial cells, and macrophages. TCs were identified morphologically by their telopodes and podoms using TEM and SEM and immunohistochemically using CD34, and vascular endothelial growth factor (VEGF). TCs also expressed CD68. TCs formed a three-dimensional network and established direct contact with blood vessels, sprouting endothelial cells, and active macrophages, while exerting their effect through paracrine signaling. VEGF was also expressed by endothelial cells and macrophages. Matrix metalloproteinase–9 (MMP-9) was expressed by TCs, endothelial cells, and macrophages. In conclusion, the expression of VEGF by TCs, endothelial cells, and macrophages is required for the proliferation and migration of endothelial cells and vascular growth. The expression of MMP-9 by TCs, endothelial cells, and macrophages is essential for the degradation of extracellular matrix (ECM) components during neoangiogenesis. Macrophages may facilitate phagocytosis and elimination of the degraded ECM components.

## Introduction

Telocytes (TCs) are a unique type of interstitial cells. They play a critical role during tissue development and hemostasis^1^. TCs consist of a cell body and telopodes (TPs), which contain focal dilatations and thin podomeres. TPs are the unique features that distinguish TCs by electron microscopy^2^. The cell body contains mitochondria, small Golgi apparatus, and smooth and rough endoplasmic reticulum^3,4^. Podoms contain endoplasmic reticulum, mitochondria, caveolae, and secretory vesicles^4^.

Angiogenesis is a physiological process through which blood vessels grow. During embryogenesis, two mechanisms of angiogenesis occur: sprouting angiogenesis and intussusceptive angiogenesis. The latter occurs during splitting of the blood vessels in current vascular tissue. Sprouting angiogenesis is a well-known type in which endothelial sprouting occurs in response an angiogenic stimulus (vascular endothelial growth factor; VEGF). Sprouting is involved in the angiogenesisof tissues devoid of blood vessels. Endothelial sprouting requires sequential steps, including enzymatic degradation of capillary basement membrane, endothelial cell (EC) proliferation, directed migration of ECs, tubulogenesis, vessel fusion, vessel pruning, and pericyte stabilization^5^.

The role of TCs angiogenesis is described in many tissues and organs during development and tissue repair^6,7^. They express VEGF, which facilitates angiogenesis^2^. The current study aims to investigate the role of TCs in angiogenesis during embryonic development of quail bird using immunohistochemistry (IHC), transmission electron microcopy (TEM), and scanning electron microscopy (SEM).

## Materials and methods

### Sample collection

Fertile quail (*Coturnix japonica*) eggs were used in the current study. The source of the eggs was the Farm of Department of Histology, Faculty of Veterinary Medicine, South Valley University, Qena, Egypt. The egg incubation protocol was carried out according to Soliman^8^. Eggs were incubated at 37.5 °C at a relative humidity 65%. Quail embryos were collected from days 5 and 8 (five for each day). The eggs were kept at − 20 °C for 4 h and were opened from the wide end, with embryos carefully excised from the egg shells. The embryos were placed on a Petri dish and fixed after washing with saline 0.9% NaCl. Sample collection was conducting in accordance with the guidelines of the Institutional Ethical Committee of Veterinary Medicine, South Valley University, Egypt, and following the Egyptian animal laws.

### Preparation of paraffin sections

*Preparation of paraffin sections was performed in accidence with*^9^*.* Embryos were fixed in Bouin’s fixative (Table 1) for 24 h^10^. Samples were dehydrated by ascending grades of alcohol, cleared by methyl benzoate (Table 1), impregnated, and embedded in paraffin wax (Table 1). Paraffin sections were cut using a Richert Leica RM 2125 microtome (Germany). Paraffin sections were cut at the neck region and stained by hematoxylin and eosin (H&E)^11^. The stained sections were examined using a Leitz Dialux 20 microscope. Photos were taken using a Canon digital camera (Canon Powershot A95).

**Table 1:** The processing time of the samples in paraffin embedding techniques.

Age process	5d	8d
**1-Fixation**		
1-F A-NBF	8 h	13 h
B_Bouin’s solution	1/2 h	1/2 h
**2-dehydration**		
Alcohol70%I	2 h	2 h
Alcohol 70%II	2 d	2d
Alchol70%III		
Alchol80%	1 h	2 h
Alchol90%	1 h	2 h
Alchol100%	1/2 h	1/2 h
Alchol100%	1/2 h	1/2 h
**3-clearing with methylebenzot**		
MB I	1 h	1 h
MB II	12 h	12 h
MB III	12 h	12 h
**4-embedding in paraffin**		
P I	2 h	2 h
P II	2 h	2 h
PIII	4 h	4 h

*NBF* neutral buffer formalin, *h* hours, *d* days, *MB I* methyl bonzoate1, *MB II* methyl benzoate II, *P I* paraffin I, *P II* paraffin II, *P III* paraffin III.

### IHC

#### IHC staining using (CD34), CD68, and matrix metalloproteinase–9 (MMP-9)

Samples were fixed in Neutral buffered formalin NBF (Table 1). The detection of antigen localization was carried out using a combination of the avidin–biotin complex technique^12^ and the solution of the Ultra Vision Detection System (antipolyvalent, horseradish peroxidase [HRP]/3,3′-diaminobenzidine [DAB] manufactured by Thermo Fisher Scientific TP-015HD). Procedures were performed according to the manufacturer’s instructions^13–15^. The Richert Leica RM 2125 microtome was used to cut 5-µm paraffin sections. The sections were dewaxed by xylene, hydrated by ascending grades of alcohol, and washed by phosphate-buffered saline (PBS; pH 7.4) (Table 2) three times for 5 min. Hydrogen peroxide was applied to the sections at room temperature to bock endogenous peroxidase activity. The sections were washed by running tap water for 10 min. A 10-mm sodium citrate buffer (pH 6.0; Table 2) was used at 95–98 °C in a water bath for 20 min for antigen retrieval. Subsequently, the slides were cooled for 20 min at room temperature and washed by PBS (pH 7.4) three times for 5 min. To prevent nonspecific background staining, Ultra V block was used at room temperature and applied for only 5 min and not more than 10 min to avoid artifact. The primary antibody (Table 3) was applied on the sections overnight at 4 °C. The sections were rinsed using PBS (pH 7.4) three times for 5 min. The biotinylated secondary antibody (goat antipolyvalent, anti-mouse immunoglobulin G [IgG] + antirabbit IgG; Thermo Fisher Scientific, UK; Lab Vision Corporation; Table 3) was applied on sections at room temperature for 10 min. The sections were rinsed by PBS (pH 7.4) three times for 5 min and incubated using streptavidin–peroxidase complex (Thermo Fisher Scientific, UK; Lab Vision Corporation, USA) for 10 min at room temperature. Visualization of the bound antibodies was performed by incubation in a humid chamber at room temperature for 5 min using a mixture of one drop of DAB and chromogen to 2 mL of DAB plus substrate. The sections were stained by a counterstain, Harris hematoxylin, for 30 s and dehydrated using ethanol and isopropanol I and II, cleared in xylene, and covered by dibutylphthalate polystyrene xylene (DPX). Immunohistochemical staining was analyzed using a Leitz Dialux 20 microscope with the Canon Power Shot A95 digital camera.

**Table 2:** Components of the fixative.

Fixative	Components	Amount
Karnovsky Fixative	Paraformaldehyde, 25% freshly prepared	10 ml
	Glutaraldehyde 50%	10 ml
	Na-Phosphate buffer (0.1 M, pH 7.4)	50 ml
	Distilled water	30 ml
Na-Phosphate buffer (0.1 M, pH 7.4)	Solution A	
	Na2HPO4 2H2O	17.02 gm
	Distilled water	600 ml
	Solution B	
	NaH2PO4 H2	6 gm
	Distilled water	200 ml
	Using solution	
	Solution A	580 ml
	Solution B	219 ml
Citrate-buffer (pH 6.0)	Solution A	
	Citrate C6H8O7 H2O	21 g
	Distilled water	1 L
	Solution B	
	Sodium citrate Na3C6H5O7 2H2O	29.41 g
	Distilled water	1 L
	Using solution	
	Solution A	9 ml
	Solution B	41 ml
	Distilled water	Add 500 ml

**Table 3:** Identity, sources, and working dilution of antibodies used in immunohistochemical studies.

Target	Primary antibody supplier	Origin (catalog no)	Dilution	incubation	Antigen retrieval	secondary antibody-incubation time
CD34	MOUSE ANTI CHICKEN CD34 (Bio rad)	MOUSE ANTI CHICKEN CD34 Monoclonal Antibody (Clone: AV138) (Cat.no MBS224490)	1:100	Over night	boiling in citrate buffer (pH 6.0), 20 min Goat	Goat anti-Mouse IgG (H + L) Secondary Antibody Catalog # 31,569 Dilution ; 1:100 One hour at room temperature
VEGF	Rabbit anti -VEGF (Invitrogen by Thermo Fisher Scientific Waltham, MA, USA))	Rabbit VEGF Polyclonal Antibody (clone: RB-222-P0) (Cat.no PA1-21,796)	1:100	Overnight	boiling in citrate buffer (pH 6.0), 20 min	Goat anti-rabbit secondary antibody (cat. no. K4003, EN Vision System Horseradish Peroxidase Labelled Polymer; Dako) Ready to use 30 min at room temperature
CD68 (Macrophage Marker) Ab-3 (Clone KP1)	Mouse Anti-CD68 ***Thermo Fisher Scientific Lab Vision Corporation, Fremont, USA***	Mouse Monoclonal Antibody **Cat. #MS-397-R7**	1:100	Overnight	boiling in citrate buffer (pH 6.0), 20 min	
MMP9 / Gelatinase B	Rabbit anti-MMP9 **LifeSpan BioSciences**	Rabbit Polyclonal antibody Catalog # LS-C31757	1:100	Overnight	boiling in citrate buffer (pH 6.0), 20 min	

Antibodies used that showed reactivity in Avian species.

#### Immunohistochemical procedures of VEGF

Two-step immunohistochemical staining procedures using the DAKO EN Vision System and HRP peroxidase were applied^16^, Paraffin sections 5-µm thick were cut using the Richert Leica RM 2125 microtome. The sections were dewaxed, rehydrated, and washed by PBS (pH 7.4) three times for 5 min. Blocking of the endogenous peroxidase was carried out using drops of 3% hydrogen peroxide in methanol for 20 min at room temperature. The section was thoroughly rinsed using running tap water for 10 min. Antigen retrieval was performed by applying 10-mm sodium citrate buffer (pH 6.0; Table 2). The sodium citrate buffer was heated in a water bath for 20 min to 95–98 °C followed by cooling for 20 min at room temperature. Sections were rinsed for 5 min by PBS (pH 7.4) three times. Blocking nonspecific background staining was carried out using drops of blocking serum (DAKO) to cover the sections for 5 min at room temperature. The primary antibody was incubated with the sections. The antibodies were successfully used in avian species^17^. Table 3 lists the identity, sources, and the working dilutions of the antibodies used in the immunohistochemical technique. The slides were washed in by PBS (pH 7.4) three times for 5 min and incubated with secondary antibody at room temperature for 30 min. The slides were washed again with PBS (pH 7.4) three times for 5 min and incubated for 5–10 min at room temperature with DAB and substrate chromogen, which revealed a brown color at the antigen site. The counterstain, Harris hematoxylin, was applied for 30 s. The sections were dehydrated by ethanol alcohol 90% and 100% II, cleared in xylene, and covered using DPX. Immunohistochemical stained sections were examined using the Leitz Dialux20 microscope provided with the Canon PowerShot A95 digital camera.

Negative controls were performed using the same procedures, except using the primary antibodies.

### Preparations of resin embedding samples^18^

Resin-embedding technique was performed using Karnovsky’s fixative^19^. The fixative was prepared as follows: 10 mL of 25% paraformaldehyde, 10 mL of 50% glutaraldehyde, 50 mL phosphate buffer, and 30 mL distilled water. Five samples were used for each age (i.e., days 5 and 8 of incubation). The neck skin was carefully excised and trimmed to a small-sized length of 2.0 to 3.0 mm. Karnovsky fixative (Table 2) was used at 4 °C overnight. The samples underwent postfixation by osmium tetroxide, dehydration, impregnation in a mixture of alcohol/resin and pure resin, resin embedding, and crystallization in an oven at 60 °C. Semithin sections were taken at 1 μm using an ultramicrotome Ultracut E (Reichert-Leica, Germany) and stained with toluidine blue^20,21^ and Periodic acid–Schiff (PAS)^22^. Staining of semithin sections was performed after dissolving the resin using a saturated alcoholic solution of sodium hydroxide. The stained sections were examined by a Leitz Dialux 20 microscope and a Canon digital camera (Canon PowerShot A95).

### TEM

Ultra-thin sections (60 nm) of resin-embedded samples at embryonic days 5 and 8 were cut using the Reichert ultra-microtome. The sections were stained using uranyl acetate followed by lead citrate for 15 min for each stain. The stained grids were examined using a JEOL100CX II transmission electron microscope at the central laboratory of South Valley University, Egypt.

### SEM

The samples were fixed in Karnovsky’s fixative and washed. Sample washing was performed using the Na–phosphate buffer (pH 7.3) for four times at 15 min. Samples were postfixed in 1% osmic acid in 0.1 M Na–phosphate buffer for an additional 2 h at room temperature and washed in Na–phosphate buffer. The samples were dehydrated by ascending grades of alcohol (50%, 70%, and 90%) for 30 min at each concentration and 100% for 2 days with many changes. Samples were treated with isoamyl acetate for 2 days and dried using the critical-point drying method with the Critical Point Drying Procedure Polaron E3000 CPD apparatus (Germany). Sample coating with gold was performed using the JEOL 1100 E-ion sputtering device (Japan) and examined with a JEOL SEM (JSM 5500 LV) at 10 kV, at the central laboratory of South Valley University, Egypt.

### Coloring of TEM and SEM images

To distinguish different types of cells and structures, coloring of TEM and SEM images was performed using the Photo Filter 6.3.2 program. The black and white image was colored using adjust then color balance to change the color to a desirable colors and degrees. Coloring transfer was performed by the stamp tool located at the right panel. Changing color was performed again using adjust then color balance to color another cell or structure. The methods used were previously described by many authors^2,4,23–31^.

### Ethical approval

The National Ethics Committee of South Valley University and veterinary authorities in South Valley University Province, Egypt, approved the method of this study. ‘All procedures were performed in accordance with the relevant guidelines and regulations^32^. Arrival guidelines the study was carried out in compliance with the ARRIVE guidelines^33^.

## Results

**Figure 1:**
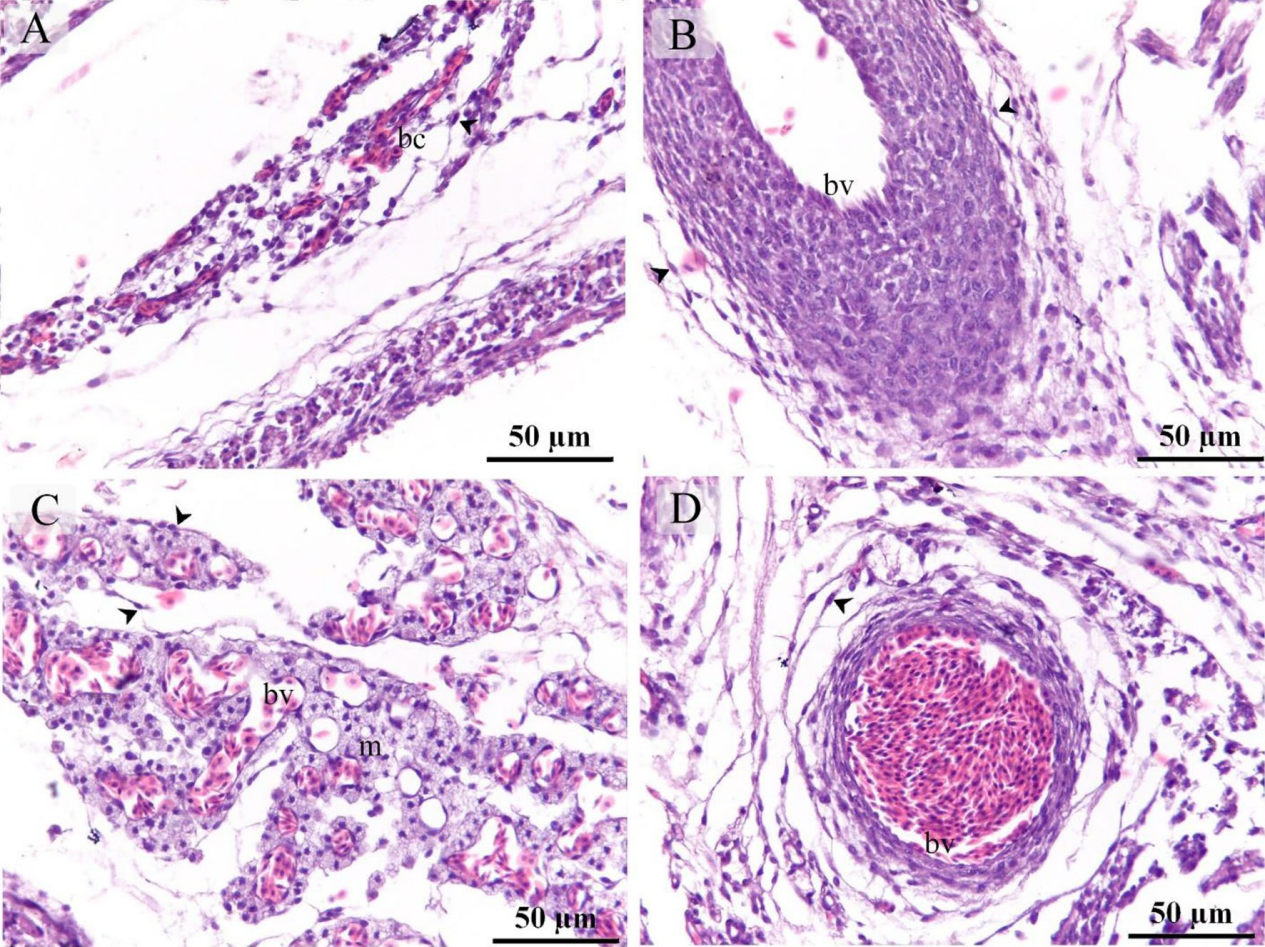
Recognition of perivascular TCs. Paraffin sections of the neck skin of day 8 quail embryos stained by H&E. (**A**) TC (arrowhead) was located around the blood capillary (bc). (**B**,**D**): TCs (arrowheads) were located around the blood vessels (bv). (**C**): TCs (arrowheads) were closely related to the vascular plexus. Note that the macrophage (m) had a vacuolated cytoplasm.

**Figure 2:**
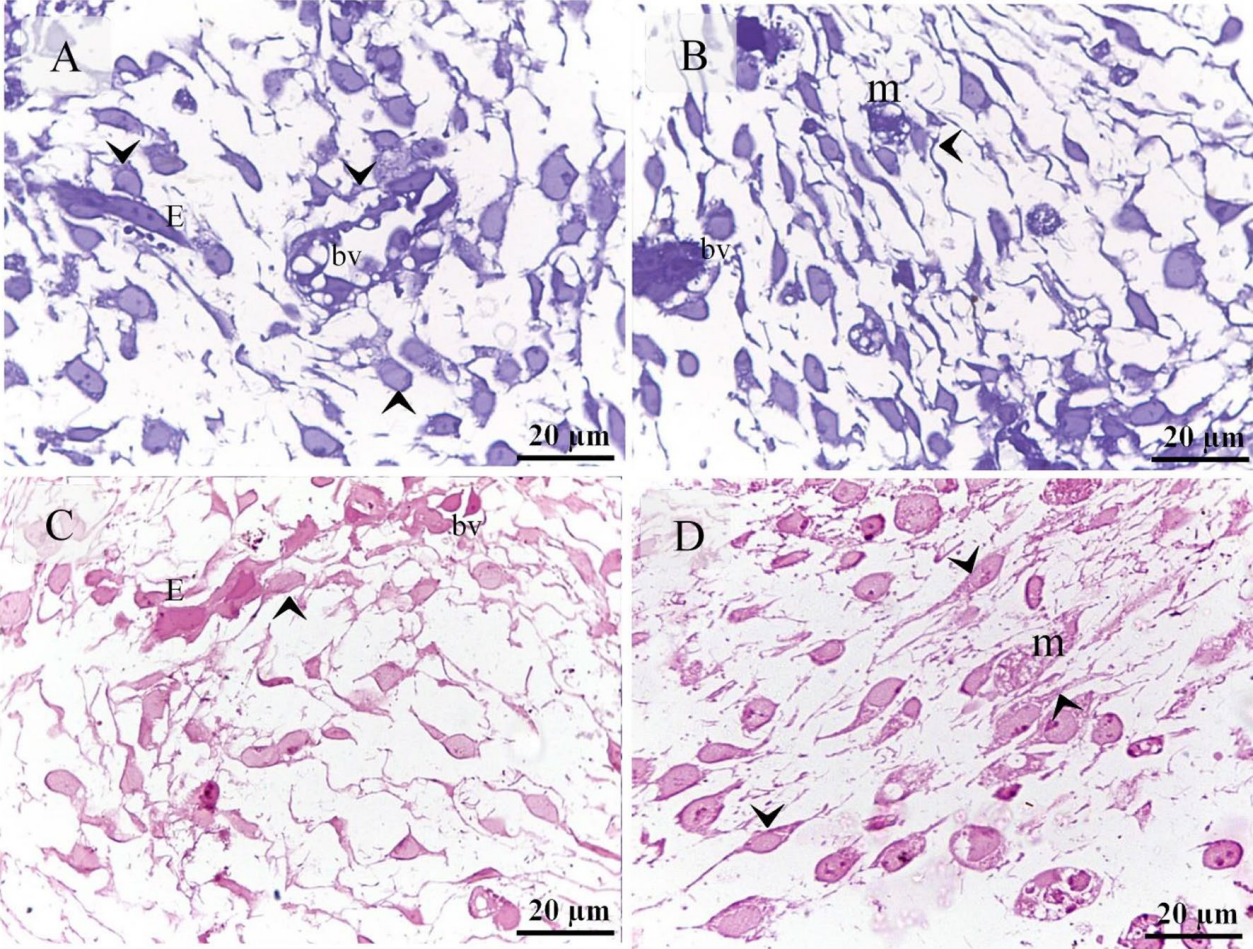
Recognition of perivascular TCs using semithin sections. Semithin sections of the neck skin of day 5 quail embryos stained by toluidine blue (**A**,**B**) and PAS (**C**,**D**). (**A**,**C**) TCs (arrowheads) located around sprouting endothelial cells (E) and blood vessel (bv). (**B**,**D**) TCs (arrowheads) located around active macrophages (m), which exhibited vesicular cytoplasm. Note the blood vessel (bv).

**Figure 3:**
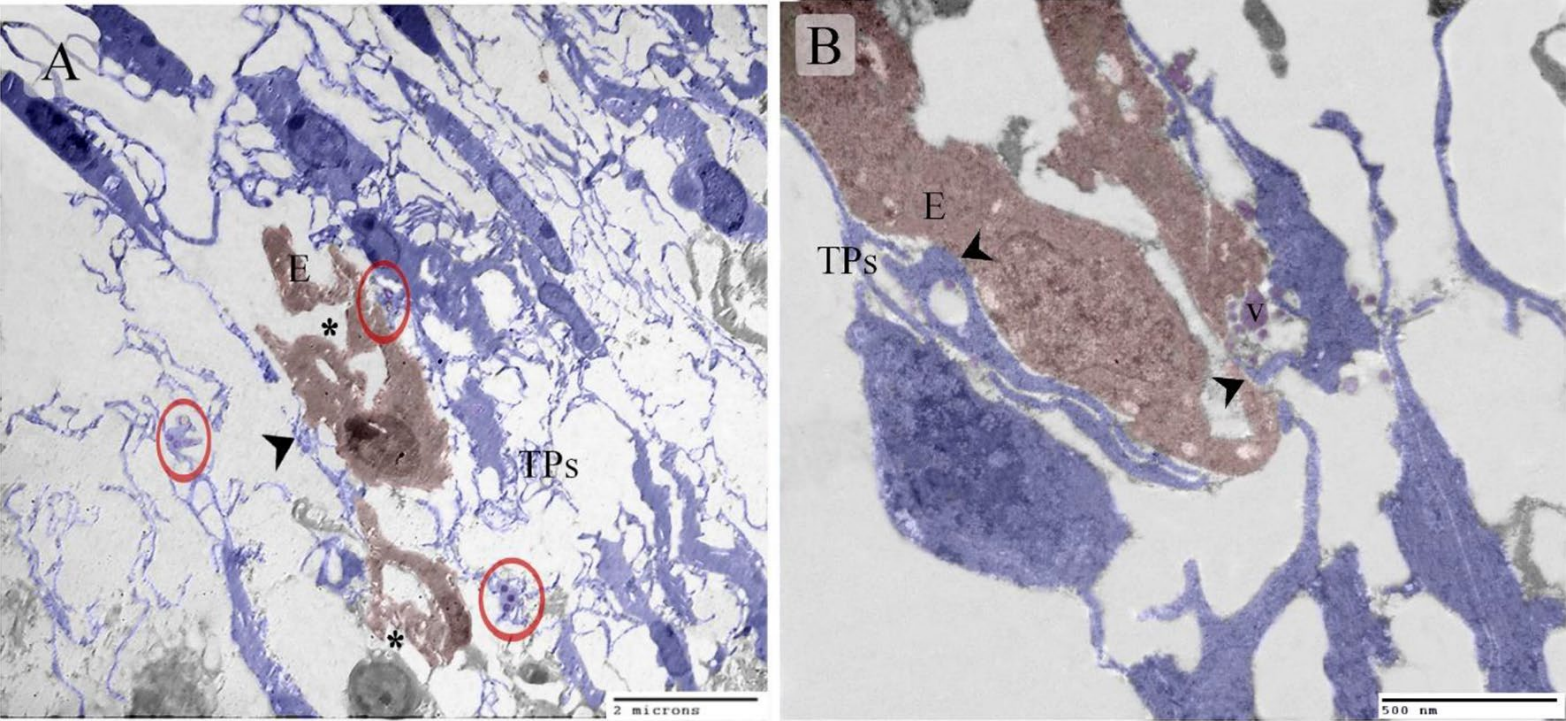
Identification of TCs using TEM. Ultrathin sections of the neck skin of day 5 quail embryos. (**A**) TCs (blue colored) identified by TPs and podoms (red circles). TCs formed an extensive 3D network. Note the sprouting endothelial cells (brown colored) that occurred after constriction (asterisk). TCs established contact with the endothelial cells (arrowhead). (**B**) TCs (blue colored) established contact with the endothelial cells (arrowhead). Note the secretory vesicles (v).

**Figure 4:**
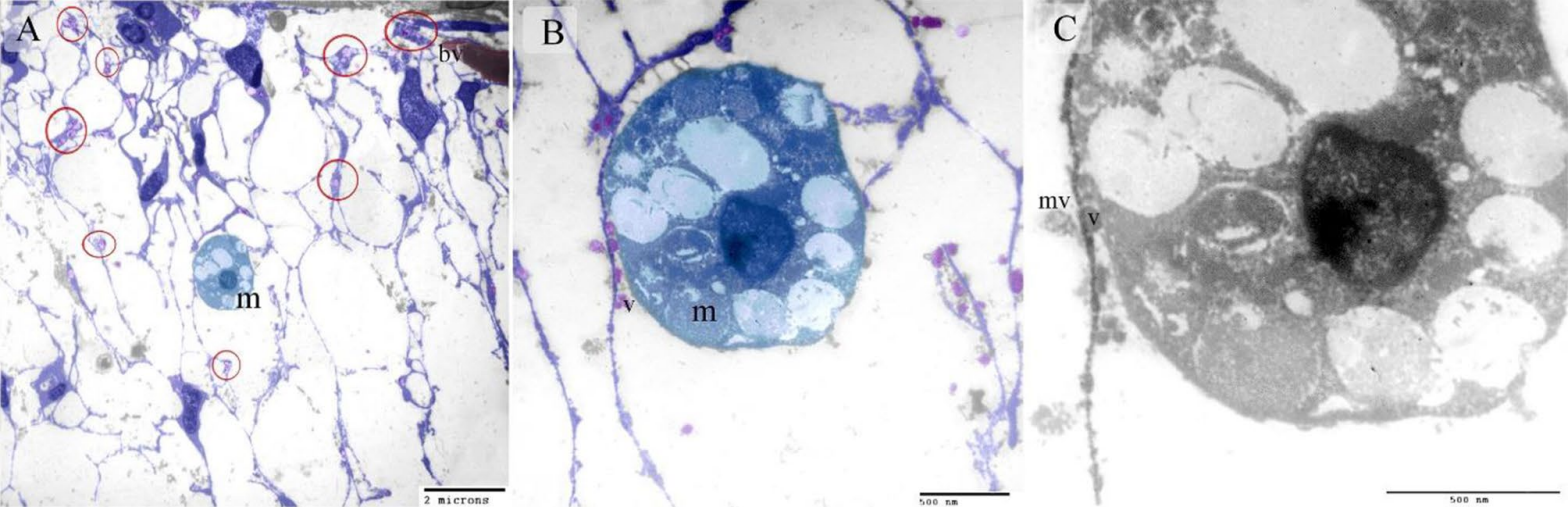
Identification of TCs using TEM. Ultrathin sections of the neck skin of day 8 quail embryos. (**A**,**B**) TCs (blue colored) identified by TPs and podoms (red circles). TCs formed an extensive 3D network. Note the TPs connected to the macrophage (m) and secretory vesicles (v). (**C**): physical contact was observed between TPs and macrophage (arrows) that had phagosomes (p) contained materials of different stages of digestion and lipid inclusions (l). Note secretory vesicles (v) and multivesicular body (mv).

**Figure 5:**
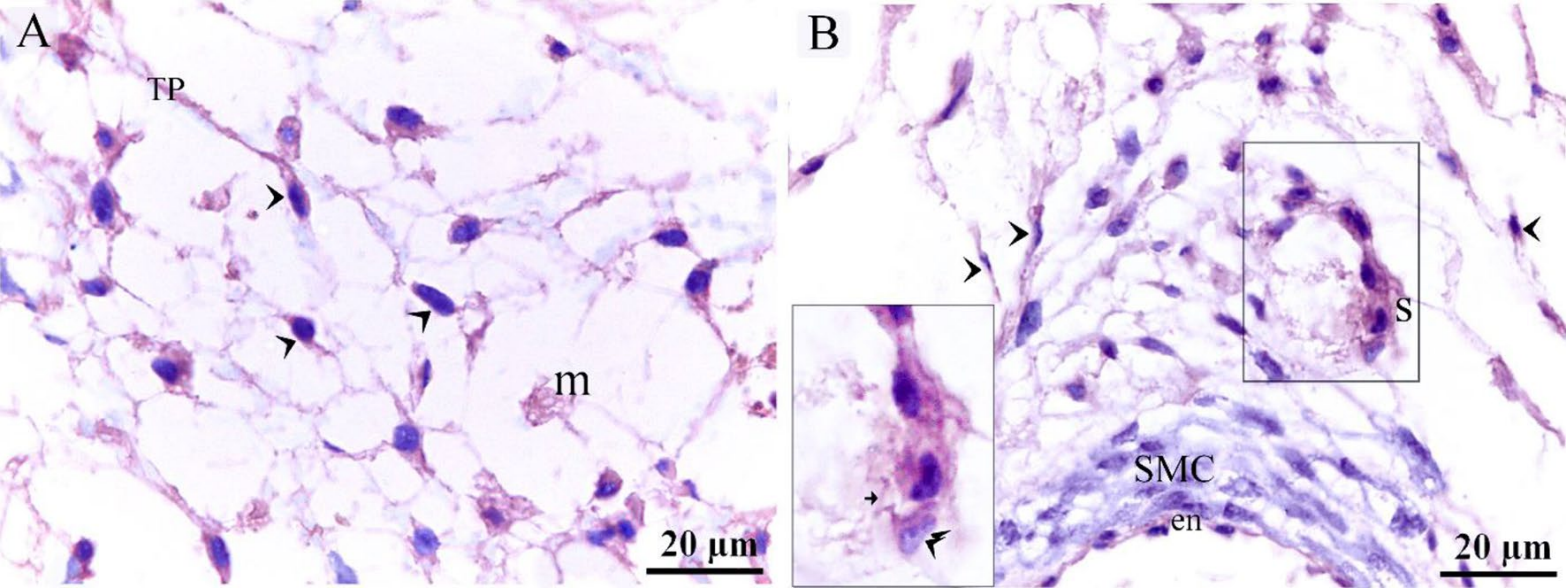
Immunohistochemical staining of the neck skin of quail embryos at day 8 using CD34. (**A**) CD34 + ve TCs (arrowheads) located around the macrophages (m). (**B**) CD34 + ve TCs (arrowheads) located around the blood vessel that consisted of endothelial cell (en) and smooth muscle cell (SMC). CD34 + ve TCs (double arrowheads) were also located around the sprouting endothelial cells (s).

**Figure 6:**
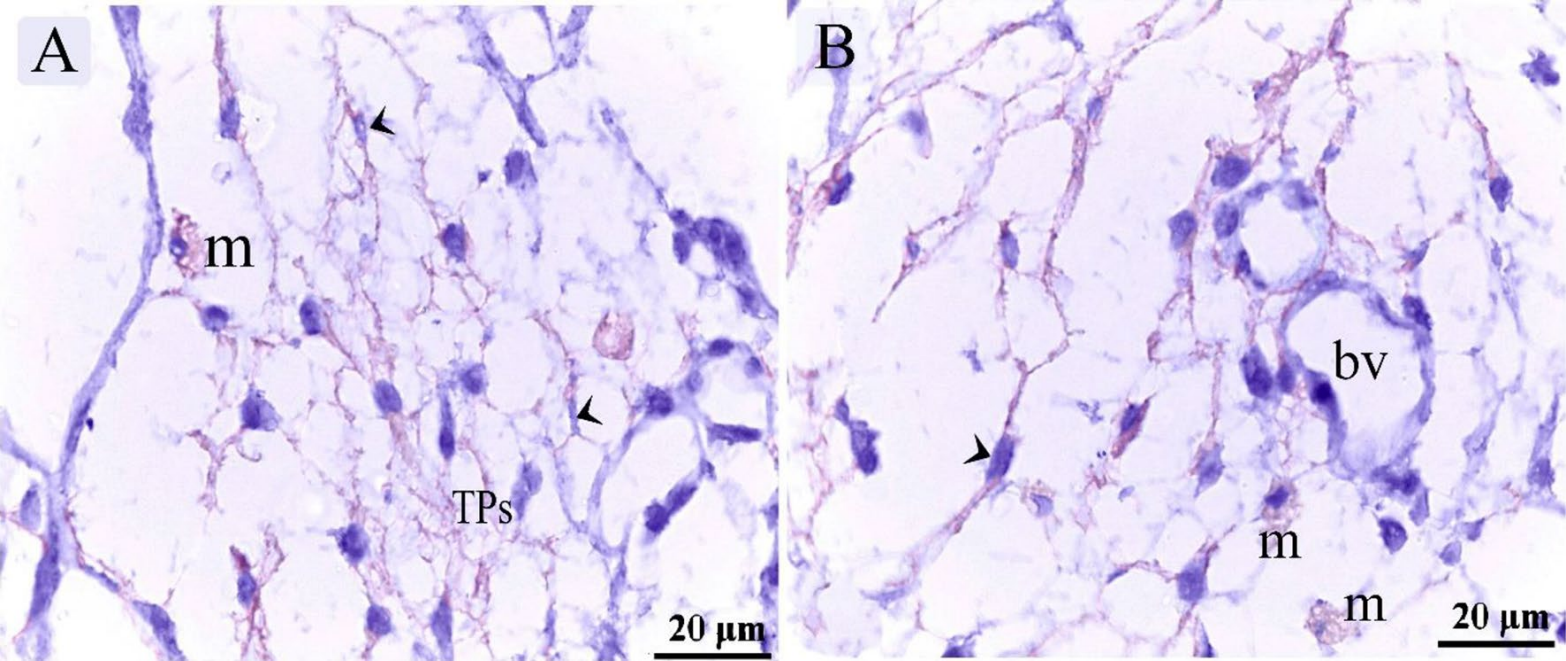
Immunohistochemical staining of the neck skin of quail embryos at day 8 using VEGF. (**A**) VEGF + ve TCs (arrowheads) located around the macrophages (m). (**B**) VEGF + ve perivascular TCs (arrowheads). Note the blood vessels (bv).

**Figure 7:**
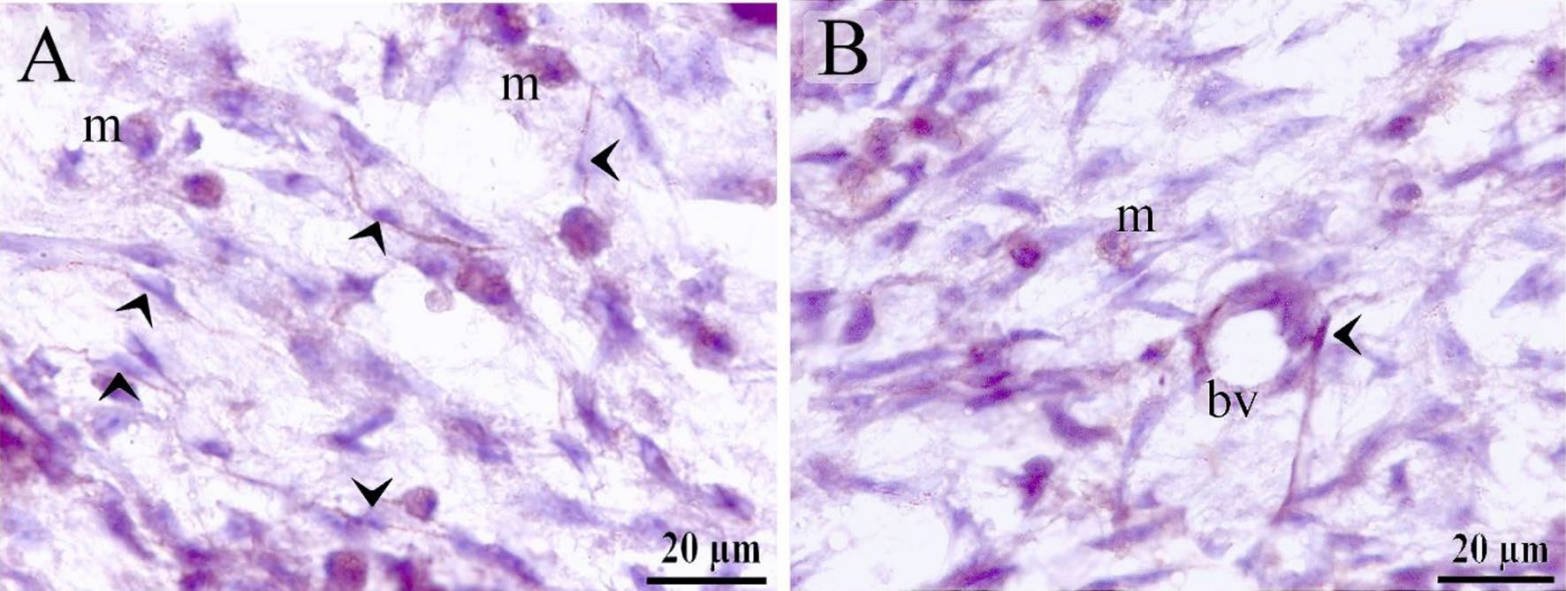
Immunohistochemical staining of the neck skin of quail embryos at day 8 using MMP-9. (**A**,**B**) TCs (arrowheads) expressed MMP-9. Note macrophages (m) and blood vessel (bv).

**Figure 8:**
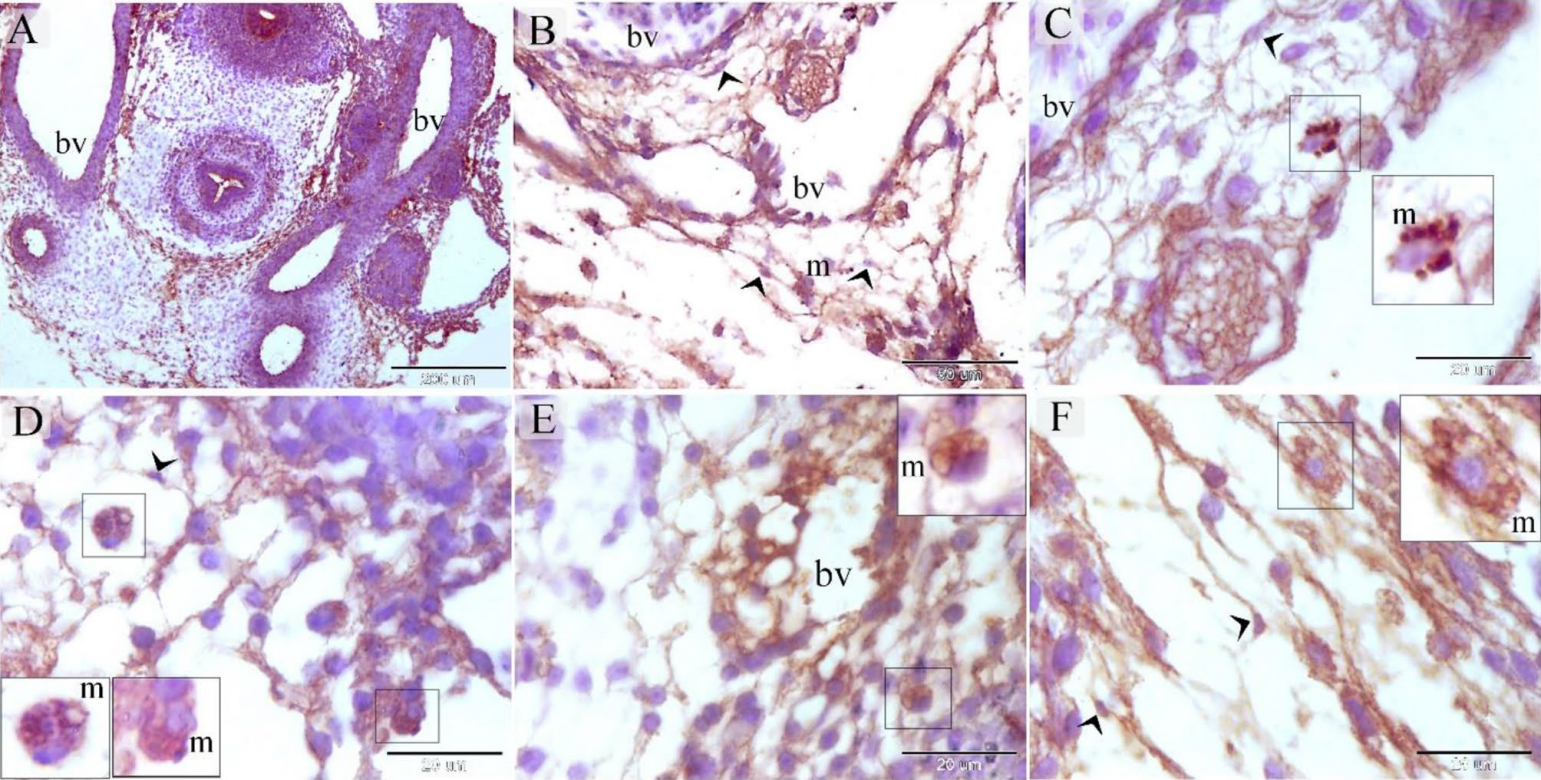
Immunohistochemical staining of the neck skin at day 5 and day 8 using CD68. (**A**) general view of the neck region at day 5 showing blood vessels (bv). (**B**,**C**,**D**) Vascular area of the neck skin of quail embryos at day 8. Note CD68 + ve macrophage (m), CD68 + ve TCs (arrowheads), blood vessel (bv). (**E**,**F**) Vascular area of the neck skin of quail embryos at day 5. CD68 + ve macrophage (m), CD68 + ve TCs (arrowheads), blood vessel (bv).

TCs were recognized around blood capillaries (Fig. 1A), large vessels (Fig. 1B), the vascular plexus (Fig. 1C) and small vessels (Fig. 1D). Perivascular TCs were also identified using semithin sections stained by toluidine blue (Fig. 2A,B) and PAS (Fig. 2C,D). They were also located in contact with sprouting ECs (Fig. 2A,C) and active macrophages (Fig. 2B,C). By TEM, TCs were identified by TPs that had distinguished podoms. TCs formed an extensive three-dimensional (3D) network (Fig. 3A). The established contact with the sprouting ECs occurred post constriction (Fig. 3A) as well as blood vessels (Fig. 3B). They released numerous secretory vesicles (Fig. 3B). TCs formed physical contact with active macrophages that had phagosomes containing materials of different stages of digestion and lipid inclusions (Fig. 4A–C). CD34 + ve TCs associated with the macrophages (Fig. 5A) and blood vessels (Fig. 5B). CD34 + ve TCs were also located around the sprouting endothelial cells (Fig. 5B). VEGF + ve TCs located around the macrophages (Fig. 6A) and the blood vessels (Fig. 6B). MMP-9 + ve TCs were detected closed to the blood vessel and macrophages and (Fig. 7A, B). TCs expressed MMP-9 (Fig. 7A,B). TCs expressed markers specific for monocyte-macrophage marker CD68 (Fig. 8A–F).

**Figure 9:**
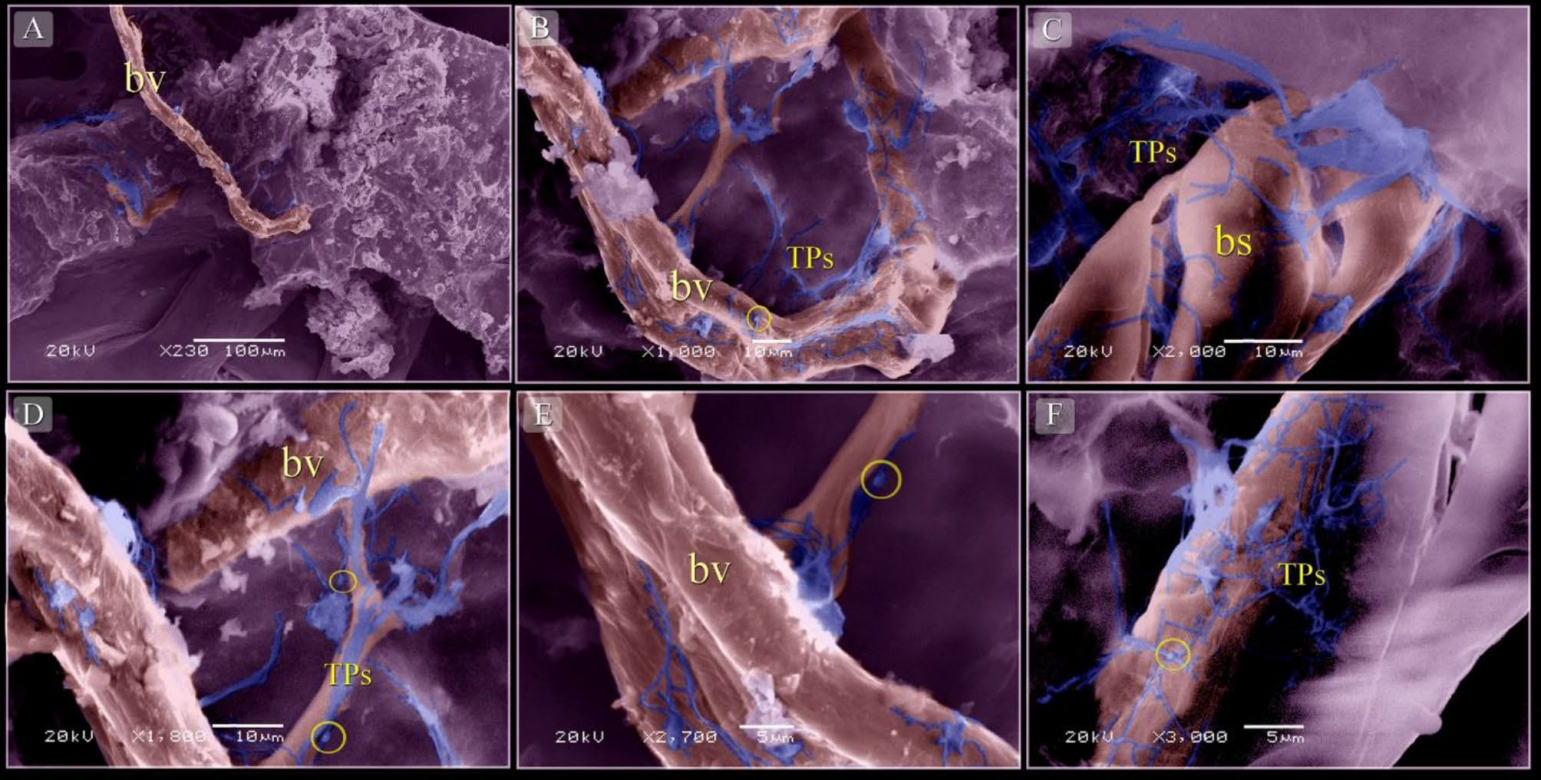
Scanned samples of the neck skin of quail embryos at day 8. (**A**,**B**,**D**–**F**) TCs (blue colored) surrounded the blood vessels (bv). Note that the TPs formed a 3D network, podoms (yellow circles). (**C**) TCs (blue colored) surrounded the blood sinusoids (bs).

**Figure 10:**
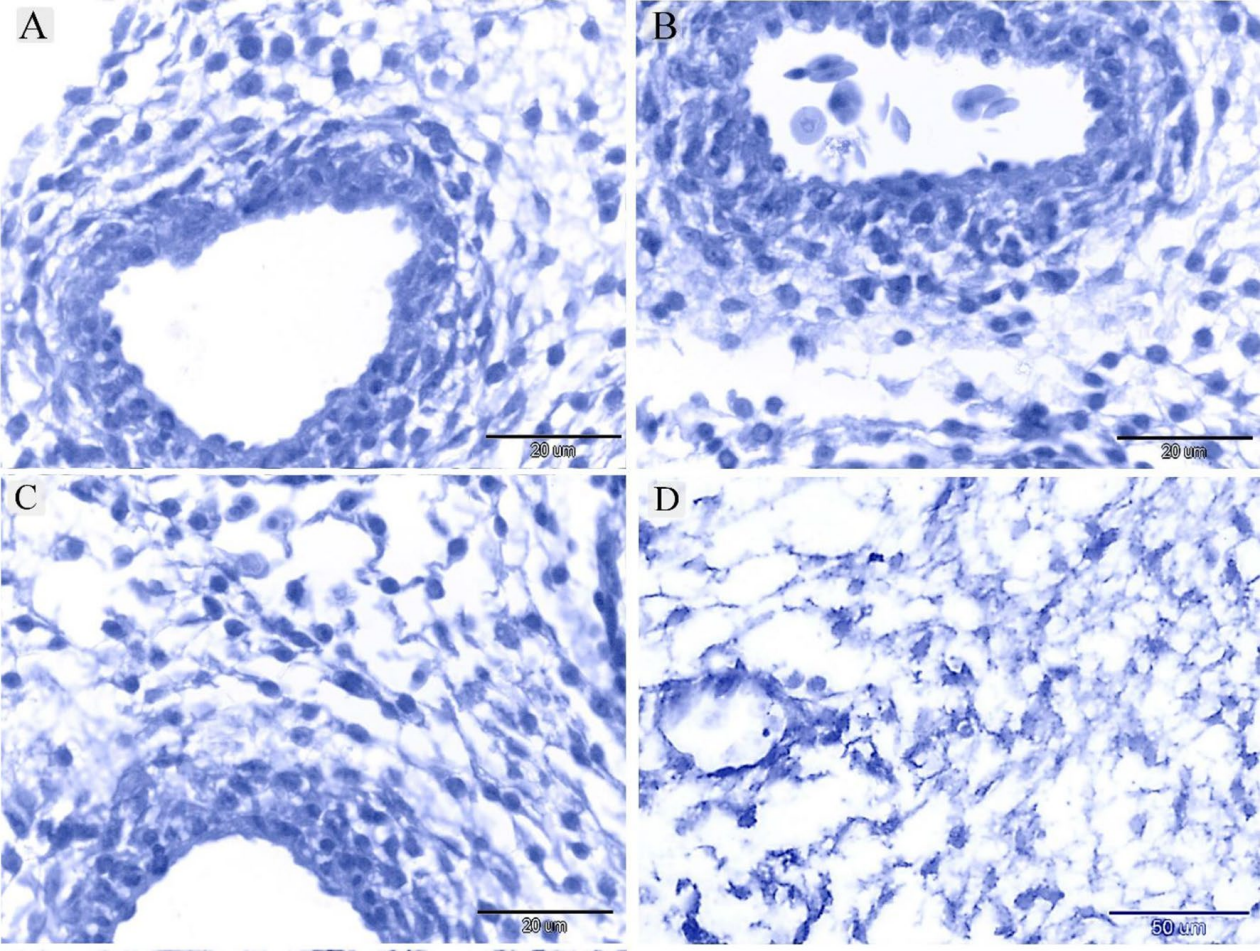
Negative control of IHC. (**A**) Negative control for CD34. (**B**) Negative control for VEGF. (**C**): Negative control for MMP-9. (**D**): negative control of the CD68.

By SEM, TCs were distinguished by TPs that formed a 3D network around blood vessels (Fig. 9A,B,D–F) and blood sinusoids (Fig. 9C). Negative control was performed for CD34 (Fig. 10A), VEGF (Fig. 10B), MMP-9 (Fig. 10C), CD68 (Fig. 10D).

## Discussion

The current study investigated the role of TCs during the early stages of angiogenesis in embryonic quail. Using TEM, SEM, and IHC, typical TCs were identified forming a 3D network in the neck skin. TCs had strong immunoaffinity for CD34 CD34 is a member protein of the transmembrane sialomucin protein family and is used for the identification of hematopoietic stem cells and non-hematopoietic progenitors, including vascular endothelial progenitors and embryonic fibroblasts, multipotent mesenchymal stromal cells, interstitial dendritic cells, and epithelial progenitors. Functional implications of CD34 are linked to cell adhesion^34^, proliferation, and inhibition of differentiation of stem or progenitor cells^35^.

Perivascular TCs formed a heterocontact with the blood vessels and release of secretory vesicles for paracrine singling. TCs established contact with sprouting ECs, which indicated neovascularization. They also expressed VEGF, which is essential for the development and growth of the circulatory system and for the regulation of proliferation and migration of vascular ECs^36^. The findings of the current study suggest the role of TCs in angiogenesis, which is supported by previous studies. They secrete VEGF and endothelial growth factor, which regulate angiogenesis and the proliferation of ECs^6^. The angiogenic role of TCs is also noted during myocardial infarction. TCs produce angiogenic microRNAs, such as let‐7e, 10a, 21, 27b, 100, 126‐3p, 130a, 143, 155, and 503^37^.

TCs communicated through direct contact and paracrine signaling with active macrophages. Avian macrophage identified by their rounded profile and contained large phagosomes with materials of different stages of digestion^38^. The relation between TCs and macrophages may reveal that TCs involved in the phagocytic activities via indirect pathway. The effect of TCs on the activation of macrophages has been studied using an in vitro coculture of peritoneal macrophages (pMACs). The authors showed that communication between pMACs and TCs occurs through heterocellular junctions and the paracrine mode, and they suggest that activation of pMACs occurs via the mitochondrial signaling pathway^39^.

MMP, or matrixins, are a type of endopeptidases. They are specialized to degrade the extracellular matrix components and other proteins and contribute to tissue remodeling^40^. Based on the biochemical properties, MMP subtypes are categorized as collagenases, gelatinases, stromelysins, and membrane-type MMPs (MT-MMPs)^41^. Gelatinases are gelatinase A (MMP-2) and gelatinase B (MMP-9)^42^. MMP-9 plays a critical role in angiogenesis, immune cell migration, activation of cytokines and chemokines, and progression and metastasis of cancer cells^43–45^. MMP-9 degrades collagen types IV, V, XIk´, XIVl´, elastin, aggrecan, link protein, decorinr, lamininn, entactin, SPARCq, myelin basic proteinm, ∞2Mn, ∞1Pli, IL-1βj, and proTNF-∞k^46,47^. In the current study, TCs exhibited proteolytic activities that had a strong immunoaffinity for MMP-9. MMP-9 plays an essential role in the steps of angiogenesis and degrades the basement membrane of capillaries. MMP-9 also promotes EC migration^43^. Vascular pruning requires post hypoxia activation of MMP-9^48^. CD68 is a sialomucin belongs to class D scavenger receptor. CD68 protein is common in late endosomes and lysosomes. Thus, CD68 protein is found in the granules of macrophages, and other cells of the mononuclear phagocyte system such as Kupffer in the liver, microglial cells in the brain, Hofbauer cell in the placenta, osteoclast in bone^49^. CD68 is also expressed by other immune cells including dendritic cells, neutrophils, basophils, and mast cells, activated T-cells and some proportion of mature B-cells as well as epithelium of renal tubules^50^. Increased CD68 expression in macrophage is associated with high vascularity^51^. In the current study, TCs expressed CD68 as well as macrophages. Stromal cells/telocytes express CD68 in the human adult trigeminal ganglion^52^. Cd68 is one of the complement Receptors that expressed by antigen presenting cells. Complement Receptors are implicated in in cell migration and phagocytosis and immune regulation. Complement regulatory proteins that expressed by antigen presenting cells have an essential role in limiting cell activation. Complement receptors cooperate with different receptors to regulate myeloid cell responses^53^.

In the current study, TCs formed 3D network at the site of neovascularization. Nehls and his colleagues noticed that the initial endothelial sprouting may not depend on pericyte. Despite of pericyte is one of the initiative types of cells that invade the nascent vascular tissue and distribute at the tip of the growing endothelial sprouts^54^. Pericyte has an essential role during angiogenesis. Development of the endothelial tube is associated with pericytes, that the endothelial sprouting act as a migration sign. pericytes are derived from the differentiation of resident mesenchymal precursors or migration from the adventitia of the adjacent vessel^55^. This process is known as pericyte-driven angiogenic process in which endothelial cells are preceded and guided by migrating pericytes^56^. pericytes may inhibit endothelial growth and migration^57,58^. Previous studies revealed a remarkable correlation between pericyte contribution and microvessel stabilization^59,60^. pericyte investment is linked to maintenance of capillary integrity in vivo^61^.Formation of vascular basal lamina and investment of the pericytes occurs at the end of the proliferative stage and the onset of the mature or quiescent stage of capillary activity^62^. endothelial cell-pericyte interactions stimulate upregulation of basal lamina-encoding genes and proteins, including fibronectin laminin, and integrins^63^. On the other hand, pericytes invasion may establish tubes and enhance the subsequent penetration of endothelial cells^64^. NG2 expressing pericyte progenitors located in close proximity to blood vessels located closed to blood vessels^65^. Thus, the reciprocal interaction between endothelial cells and pericytes has a fundamental role in angiogenic process.

In conclusion, TCs facilitate angiogenesis. They express VEGF, which promotes endothelial proliferation and migration. They also release MMP-9, which plays a critical role in the degradation of the capillary basement membrane, EC migration, and vascular pruning.

## Supplementary Information

Supplementary Information

## Data Availability

All data generated or analysed during this study are available on the author.
