# Supplementary material for: Telocytes are major constituents of the angiogenic apparatus
Source: Sci Rep. 2021 Mar 11;11:5775. doi: 10.1038/s41598-021-85166-w (PMC7952407; doi:10.1038/s41598-021-85166-w)
Supplement: Supplementary file 1 — Supplementary Information [file 41598_2021_85166_MOESM1_ESM.pdf]

Title page

Title: **Telocytes are major constituents of the angiogenic apparatus**

Author name: **Soha Abel-lateif Soliman**

Address: Faculty of Veterinary Medicine, South Valley University, Qena, Egypt
Affiliation: Department of Histology, Faculty of Veterinary Medicine, South Valley University, Qena, Egypt
E-mail address and telephone number(s) of the corresponding author: soha_soliman@vet.suv.edu.eg, +201006500848

Assignment number: EKBEAW-634

Filename: Telocytes are major constituents of the angiogenic apparatus (2)_INQ-6155334820_EKBEAW-634.docx

Primary Editor Name: Kim D

[1] **In case of any clarifications or questions please approach us at submit@enago.com**

Dear Author:

Thank you for entrusting us with your manuscript and opting for our Copyediting service.

We have edited your assignment EKBEAW-634 for language and grammar, from the aspect of fluency, and would like to share our experience in editing your manuscript.

Overall, we have made moderate revisions to the manuscript in terms of language and grammar. We have also made some sentence reconstructions and word choice changes for accuracy and enhanced clarity. We hope that the revisions meet your expectations from our service.

Please be assured that we have edited the manuscript to the best of our ability and have clarified some of our changes through remarks. As a step toward finalization, we suggest that you resolve all remarks in the main file, as this is important for successful publication.

We have added a tip on academic writing conventions specific to your manuscript in the edited file. We hope you find this tip helpful for future writing.

We wish you the very best for the successful publication of your manuscript and look forward to working with you again.

Sincerely,

Your Editor

Kim D
